# Comparison of the Use of a Physiologically Based Pharmacokinetic Model and a Classical Pharmacokinetic Model for Dioxin Exposure Assessments

**DOI:** 10.1289/ehp.8016

**Published:** 2005-08-25

**Authors:** Claude Emond, Joel E. Michalek, Linda S. Birnbaum, Michael J. DeVito

**Affiliations:** 1 National Research Council, National Academy of Sciences, Washington, DC, USA; 2 Pharmacokinetics Branch, Environmental Toxicology Division, National Health and Environmental Effects Research Laboratory, Office of Research and Development, U.S. Environmental Protection Agency, Research Triangle Park, NC, USA; 3 Air Force Research Laboratory, Brooks City-Base, Texas, USA

**Keywords:** dioxin, epidemiology, PBPK, pharmacokinetic, physiologically based pharmacokinetic model, Ranch Hand, risk assessment

## Abstract

In epidemiologic studies, exposure assessments of 2,3,7,8-tetrachlorodibenzo-*p*-dioxin (TCDD) assume a fixed elimination rate. Recent data suggest a dose-dependent elimination rate for TCDD. A physiologically based pharmacokinetic (PBPK) model, which uses a body-burden–dependent elimination rate, was developed previously in rodents to describe the pharmacokinetics of TCDD and has been extrapolated to human exposure for this study. Optimizations were performed using data from a random selection of veterans from the Ranch Hand cohort and data from a human volunteer who was exposed to TCDD. Assessment of this PBPK model used additional data from the Ranch Hand cohort and a clinical report of two women exposed to TCDD. This PBPK model suggests that previous exposure assessments may have significantly underestimated peak blood concentrations, resulting in potential exposure misclassifications. Application of a PBPK model that incorporates an inducible elimination of TCDD may improve the exposure assessments in epidemiologic studies of TCDD.

Exposure to 2,3,7,8-tetrachlorodibenzo-*p*-dioxin (TCDD) is associated with increased risk for cancer, diabetes, and reproductive toxicities in numerous epidemiologic studies (Schecter and Gasiewicz 2003). Several of these studies base exposure estimates on measurements of blood levels years after accidental or occupational exposures. Peak exposures have been estimated in these studies assuming a mono- or biphasic elimination rate for TCDD, with estimates of half-life ranging from 5 to 12 years (Hooiveld et al. 1998; Michalek et al. 2002; Steenland et al. 2001). Recent clinical studies suggest that the elimination rate of TCDD is dose dependent (Michalek et al. 2002). In experimental animals, several studies also demonstrate dose-dependent elimination (Abraham et al. 1988; Diliberto et al. 2001). In both the animal and human data, as the exposure dose increases the apparent half-life decreases, indicating an inducible elimination of TCDD.

We developed a physiologically based pharmacokinetic (PBPK) model that describes the pharmacokinetics of TCDD in rodents (Emond et al. 2004). This approach is a mathematical description of the physiologic, biochemical, and physicochemical processes involved in the pharmacokinetics of TCDD. This model, originally validated in rodents, includes a mathematical description of the aryl hydrocarbon receptor–mediated induction of cytochrome P450 1A2 (CYP1A2). In the model, the elimination rate of TCDD is dose dependent and is a function of CYP1A2 induction. Experimental evidence suggests that CYP1A2 is responsible for hepatic sequestration of TCDD (Diliberto et al. 1997) and is also one of the enzymes responsible for its metabolism (Hakk and Diliberto 2002). Thus, at low exposures, there is minimal induction and the elimination of TCDD is very slow. However, at higher exposures, induction approaches a maximum and the elimination rate is much faster. Human physiologic and biochemical parameters were incorporated into the rodent PBPK model for species extrapolation.

## Materials and Methods

In the present study a rodent PBPK model (Emond et al. 2004) was extrapolated to humans. Initial optimization of the human PBPK model used two data sets. The first data set comes from studies of U.S. Air Force veterans from Operation Ranch Hand. Veterans involved in Operation Ranch Hand were responsible for the aerial spraying of Agent Orange and other herbicides contaminated with TCDD during the Vietnam War from 1962 to 1971. We selected a subpopulation involving 343 Ranch Hand veterans and determined TCDD concentrations in blood samples collected every 5 years from 1982 to 1998 for a total of four or five samples from each veteran from this subpopulation (Michalek et al. 2003). Data from 20 randomly selected subjects from the Ranch Hand cohort subpopulation were used to optimize the human PBPK. The second set of data used to optimize the model was from Poiger and Schlatter (1986), in which a single volunteer received a single oral dose of 1.14 ng TCDD/kg and was followed for 40 days. These data were used in the optimization of the absorption and distribution processes occurring during the initial phase of the exposure.

Our assessment of the human PBPK model used an additional 10 randomly selected subjects from the Ranch Hand cohort and showed a good correlation (*r*^2^ = 0.995) between predicted blood concentrations in 1982 and measured blood concentrations in 1982 (Table 1). We also assessed the human PBPK model with a second data set. In the fall of 1997, two women presented clinical signs of TCDD intoxication (Geusau et al. 2002). After presentation of chloracne, between the spring of 1998 through 2001, 25 and 20 blood samples were collected from patients 1 and 2, respectively (Geusau et al. 2002). These women are among those with the highest TCDD blood concentrations ever measured in adults.

## Results

In the veterans of Operation Ranch Hand, TCDD blood concentrations were first determined starting in 1982 (Michalek et al. 1996, 2002). The exposure occurred between 1962 and 1971, with a typical tour of duty lasting only a year. Peak blood concentrations were assumed to occur at the time of discharge from Vietnam. We documented the time of discharge for each veteran in the Ranch Hand cohort, and used these individual data in the back calculation for this study. TCDD blood concentrations were determined at four or five time points for each Veteran starting in 1982. For each TCDD measurement we used data on body weight and height for each individual to estimate the body mass index for each veteran. We used the body mass index to estimate size of the adipose tissue compartment at the time of TCDD measurement for each individual based on the approach of Deurenberg et al. (1991). We estimated peak TCDD blood concentrations for each individual with the PBPK model using their individual data on blood concentrations, adipose tissue mass, and the time of discharge from Vietnam. We also estimated peak blood concentrations using a classical one compartment pharmacokinetic model with a first-order elimination. The classical model assumed a TCDD half-life of 8.7 years and used the TCDD blood concentrations at 1982 (Michalek et al. 1996) and the time of discharge as inputs into the model to estimate peak blood concentrations.

**Figure 1:**
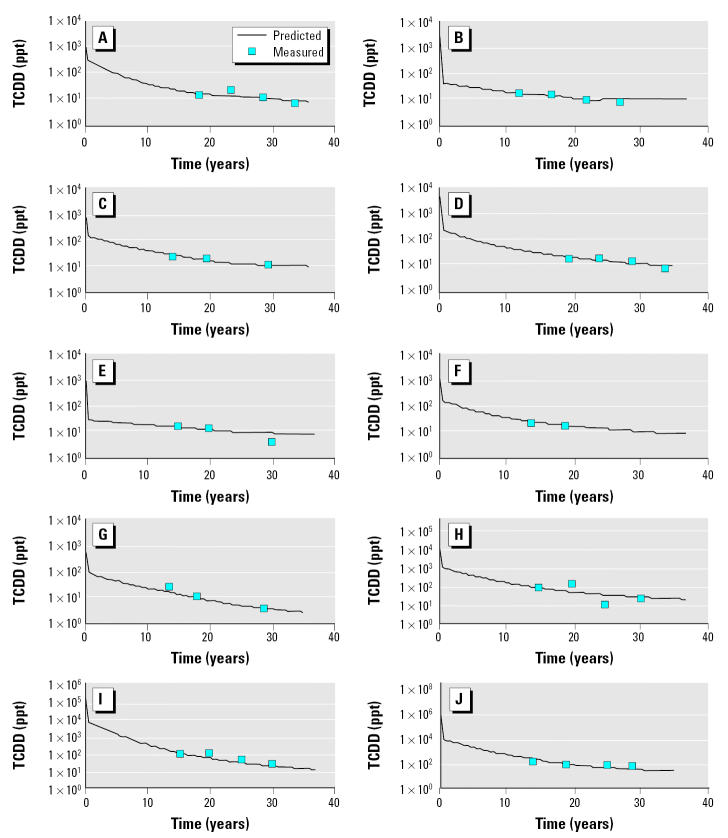
Model predictions of TCDD blood concentration in 10 veterans (*A–J*) from the Ranch Hand cohort.

In 1982, the range of blood concentrations from 10 randomly chosen subjects, shown in Table 1, was approximately 16-fold, from 12.7 to 209 ppt. We used a classical pharmaco-kinetic approach; peak blood concentrations ranged approximately 12-fold, from 53 to 640 ppt (Table 1). Minor differences in the ranking and range of TCDD blood concentrations occur when comparing estimated peak concentrations using the one compartment classical pharmacokinetic model to blood concentrations measured in 1982. When using the PBPK model to estimate peak blood concentrations, we found a much larger range in exposures and a significant difference in the exposure rankings (Table 1). The PBPK model estimates that peak blood concentrations at the time of discharge range > 250-fold, from 138 to approximately 40,000 ppt. This large difference is due to the inclusion of a dose-dependent elimination rate in the PBPK model. At the lower exposures, the half-life of TCDD is > 10 years, and at the higher exposures the half-life is only weeks. Models fits to these data are presented in Figure 1.

**Figure 2:**
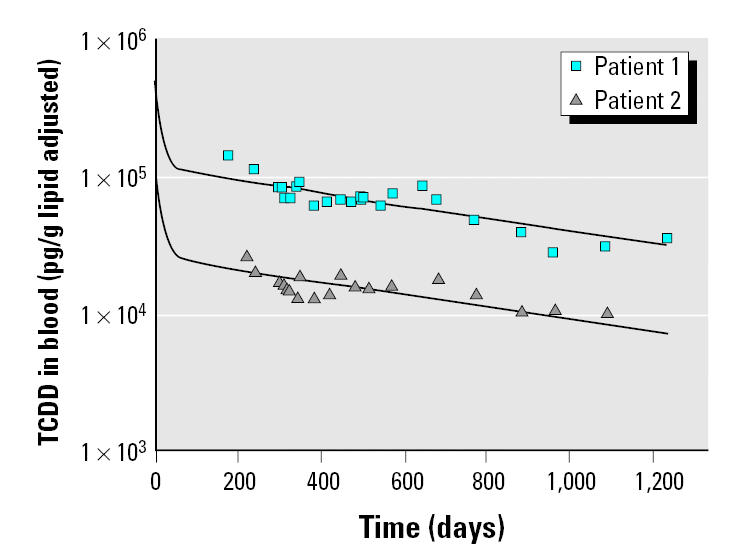
Time course of TCDD in blood (pg/g lipid adjusted) for two highly exposed women (patients 1 and 2). Symbols represent measured concentrations, and lines represent model predictions. These data were used as part of the model evaluation (Geusau et al. 2002).

The model predictions show good correlations with the measured blood concentrations in the two highly exposed women (Figure 2). The model predicts a rapid decrease in the blood concentrations during the distribution phase of the first few months of exposure, followed by an elimination that appears first order at these exposures because of maximal induction of TCDD sequestration metabolism. The elimination rates in these women suggest that the overall half-life of TCDD during the first 2 years of exposure is < 3 months. In the first blood samples collected from these women, the concentrations of TCDD were 144,000 and 26,000 ppt (lipid adjusted) in patient 1 and 2, respectively (Geusau et al. 2002). The PBPK model estimates that initial blood concentrations may have been as high as 507,000 ppt and 87,000 ppt (lipid adjusted) in patients 1 and 2, respectively. Based on this model, maximum CYP1A2 induction occurs at blood concentrations of approximately 1,250 ppt (lipid adjusted). Measured levels of TCDD in the women were approximately 20–100 folds higher than the blood concentrations that are predicted to be at maximal induction (Geusau et al. 2002).

## Discussion

Studies on the elimination of TCDD have examined cohorts many years after the exposures and suggest that the half-life approaches a decade. However, these studies did not examine the initial elimination of TCDD immediately after high-level exposures. The high concentration predicted with the model during the first 6 months is an extrapolation of what should be the concentration at the time of initial exposure. Limited data are available to validate the model for the initial exposure period. One data set is available from Poiger and Schlatter (1986). Although these data were used in the optimization of the model, the small sample size and only a single dose level do not provide confidence that the data from Poiger and Schlatter (1986) represent the wide range of potential exposures and populations at risk.

A number of pharmacokinetic models have incorporated dose-dependent elimination of TCDD. These models use a variety of approaches to describe the dose dependency. Andersen et al. (1993) use a hyperbolic function related to receptor occupancy to describe the dose-dependent elimination. This function is modified by a species specific “fold” factor that is used to adjust the elimination rate. In rats this factor is 1 and allows for a doubling of the elimination rate; other species would have different adjustment factors. Kohn et al. (2001) also use a Hill equation for the kinetics of the metabolizing enzyme with cytosolic TCDD concentrations as the substrate concentration. TCDD is also hypothesized to be eliminated through biliary pathways after hepatocyte lysis at high exposures in the model of Kohn et al. (2001). In the models of Carrier et al. (1995a, 1995b) and Aylward et al. (2005), the elimination of TCDD is described as a function of total hepatic TCDD concentrations. The elimination of TCDD in these models is dose dependent because there is a dose-dependent sequestration of TCDD in the liver. In the present model we describe the elimination rate as a function of CYP1A2 induction. The different approaches used to describe the dose-dependent induction of TCDD elimination are due to a lack of understanding of the biologic basis of these phenomena. This uncertainty in our understanding of the elimination of TCDD indicates that caution should be used when applying any of these models to human epidemiologic studies. However, the use of dose-dependent elimination of TCDD is an important concept to consider when choosing and applying pharmacokinetic tools in exposure assessments for dioxin.

Recent studies that measured TCDD blood concentrations shortly after high-level exposure indicate that the half-life is dose dependent (Geusau et al. 2002), as do clinical studies of the Ranch Hand cohort (Michalek et al. 2002). The use of first-order elimination of TCDD could significantly underestimate past exposures, resulting in exposure misclassifications in the epidemiologic studies. Using a PBPK model that incorporates a dynamic elimination rate may provide a more accurate assessment of past exposures in the epidemiologic studies. A better understanding of the biologic basis of the dose-dependent elimination of TCDD would allow for the development of more biologically realistic PBPK models. Further validation of this model is required before use in a quantitative exposure assessment. However, a pharmacokinetic model that includes an inducible elimination should be applied when assessing past exposures to TCDD.

## Figures and Tables

**Table 1:** Comparison of initial blood concentration (*C*_blood_) determination by first-order elimination or by PBPK model in 10 Ranch Hand veterans.*^a^*

	*C*_blood_ in 1982		*C*_blood_ at the time of discharge from Vietnam	
Group	Measured (pg/g lipid adjusted)	Predicted with PBPK model (pg/g lipid adjusted)	Estimated with constant *T*_1/2_ of 8.7 years (pg/g lipid adjusted)	Estimated with a PBPK model (pg/g lipid adjusted)
Low	12.7	13.7	53	138
	16.7	20.1	44	166
	23.5	26.9	72	277
	24.6	29.5	112	587
	25.0	19.4	83	168
High	33.7	37.8	103	492
	43.8	25.5	123	197
	115.5	132.3	381	6,622
	182.3	198.3	602	40,376
	209.7	234.6	640	35,412

*T*_1/2_, half-life of TCDD in the blood.

a The model provides a good prediction of the measured blood concentrations in 1982 with a coefficient of determination of *R*^2^ = 0.995.
